# Production and Characterization of a Novel Low-Sugar Beverage from Red Jujube Fruits and Bamboo Shoots Fermented with Selected *Lactiplantibacillus plantarum*

**DOI:** 10.3390/foods10071439

**Published:** 2021-06-22

**Authors:** Chu-Min Zhao, Ting Du, Ping Li, Xin-Jun Du, Shuo Wang

**Affiliations:** 1 State Key Laboratory of Food Nutrition and Safety, College of Food Science and Engineering, Tianjin University of Science and Technology, Tianjin 300457, China; zcm3278@163.com (C.-M.Z.); tingdu@tust.edu.cn (T.D.); zoelxx@tust.edu.cn (P.L.); 2 Tianjin Key Laboratory of Food Science and Health, School of Medicine, Nankai University, Tianjin 300071, China

**Keywords:** red jujube, bamboo shoot, *Lactiplantibacillus plantarum*, fermentation, nutrient contents

## Abstract

Red jujube fruits and bamboo shoots are rich in many nutrients and have the advantage of high yield in China. However, the storage of fresh fruits is difficult, and there are no fermented products using both as raw materials. In order to develop the two raw materials into novel products and improve their nutritional value, this study reports the production and characterization of a beverage via fermentation of red jujube fruits and bamboo shoots with *Lactiplantibacillus plantarum*. *L. plantarum* TUST-232 was selected as the starter from several different strains by comparing pH value and the number of viable cells, which reached 8.91 log CFU/mL in the beverage fermented for 14 h at 37 °C with 0.3% inoculation. After fermentation, the beverage showed improvement in the contents of several nutrients and antioxidant indices, with a decrease of 44.10% in sucrose content, along with increases of 11.09%, 12.30%, and 59.80% in total phenolic content, total antioxidant capacity, and superoxide anion scavenging ability, respectively. These results indicate that *L. plantarum* fermentation of red jujube fruits and bamboo shoots could be an effective way to develop a new beverage with high nutritional value, high antioxidant capacity, and high dietary fiber content. This research provided experimental support for the development of new fermentation products with the functions of improving health and body functions.

## 1. Introduction

Red jujube (*Ziziphus jujuba* Mill), a fruit native to China, is rich in nutrients and functional components, such as vitamin C, polysaccharides, dietary fibers, carbohydrates, triterpenoid acids, flavones, and phenolic acids [1]. Bamboo shoot, the bud of Gramineae Bambusoideae, is not only delicious, but also rich in nutrients, proteins, and dietary fibers, thus being beneficial to intestinal health [2,3]. Although fruits and vegetables are rich in nutrition and beneficial to human health, their daily intake is lower for many people than that recommended by the World Health Organization [4].

Lactic acid bacteria (LAB) are usually beneficial microorganisms, with many beneficial effects on humans and animals when properly ingested [5,6]. LAB-fermented juices can not only facilitate the intake of fruits and vegetables, but also improve the sensory quality, flavor substances, safety, and shelf-life of beverages [7]. The amount of yeast and mold in the fermented juices can be kept at a very low level after storage at 4 °C for 2 or 3 weeks [8]. More importantly, LAB can release specific bioactive compounds, which are beneficial to people’s health [9]. Various studies have confirmed the role of LAB in improving nutritional value and treating intestinal diseases, such as improving the flavor and antioxidant activity, extending shelf-life, regulating the balance of intestinal microbiota, promoting the absorption of nutrients, and improving the body’s immunity [10,11]. Therefore, LAB-fermented beverages have gradually attracted the attention of researchers and gained the recognition of consumers [12]. However, to our knowledge, no fermented beverage has been developed using red jujube fruits and bamboo shoots as raw materials.

The purpose of this study was to develop and characterize a low-sugar, high-antioxidant, and fiber-rich fermented beverage of red jujube fruits and bamboo shoots by evaluating the effects of *Lactiplantibacillus plantarum* on the metabolism of sugars and organic acids, as well as the changes in antioxidant activity, total phenolic content, total flavonoid content, and dietary fiber content in the beverage. To the best of our knowledge, this is the first report on the development and characterization of a fermented beverage from red jujube fruits and bamboo shoots, and the results will provide useful information for further processing and utilization of red jujube fruits and bamboo shoots.

## 2. Materials and Methods

### 2.1. Strains and Raw Materials

*Streptococcus thermophilus* 20364 and *Lactobacillus delbrueckii* subsp. *bulgaricus* 20247 were obtained from the China Center of Industrial Culture Collection, CICC^®^. *Lactiplantibacillus plantarum* TUST-232, *L. plantarum* TUST-354, and *L. plantarum* TUST-392 were provided by the State Key Laboratory of Food Nutrition and Safety, Tianjin University of Science and Technology. Modified Chalmers media (MC) (Haibo, Qingdao, China) was used for the culture of *S. thermophilus*, and the other bacteria were cultured using De Man, Rogosa, Sharpe (MRS) media (Haibo, Qingdao, China).

After washing, the red jujube fruits were first boiled to preliminarily remove bitterness and astringency and then ninefold diluted with distilled water. Next, 0.1% (*m/v*) pectinase (50,000 U/g) and 0.05 (*m/v*) cellulase (100,000 U/g) were added to hydrolyze the red jujube fruits at 50 °C for 90 min to improve the pulp yield [13]. After removing the shell and cleaning, the bamboo shoot was mixed with fourfold distilled water before crushing. In order to improve the color of the mixture, the pretreated red jujube pulp and the bamboo shoot pulp were mixed at a ratio of 4:6, followed by grinding with a colloidal mill (Taichi, Shanghai, China) and a high-pressure homogenizer (Shenlu, Shanghai, China). The backlash of the colloidal mill was 1–2 mm. After a significant reduction in particle size, the mixture of the red jujube fruits and the bamboo shoots (R–B mixture) was further ground with a homogenizer and treated three times under 25–30 MPa. Finally, the R–B mixture was autoclave-treated at 105 °C for 15 min to obtain the mixed juice of the red jujube fruits and the bamboo shoots (R–B mixed juice) and then stored in a refrigerator (2–4 °C) until further use.

### 2.2. Strains Screening

The fermentation was performed in conical flasks, and the most suitable fermentation strain was selected by comparing the fermentation ability of different starter cultures to the R–B mixed juice, including mixed *L. delbrueckii* subsp. *bulgaricus* and *S. thermophilus* (TUST-BS for short), *L. plantarum* TUST-232, *L. plantarum* TUST-354, and *L. plantarum* TUST-232.

Firstly, the R–B mixed juice was fermented separately by all the starter cultures under different inoculation volumes (0.03%, 0.06%, 0.10%, 0.20%, 0.30%, and 0.40% with separate trials) (TUST-BS: the 2:1 ratio of *L. delbrueckii* subsp. *bulgaricus* and *S. thermophilus*) and fermentation temperatures (30 °C, 34 °C, 37 °C, and 40 °C with separate trials) for 24 h to screen the fermentation conditions of strains initially via measuring the pH values and viable cell counts. Then, according to the results of inoculation volumes and fermentation temperatures, all the starter cultures (the inoculation volume of 0.30%, about 7 log CFU/mL) were inoculated separately into the R–B mixed juice and cultured at 37 °C for 24 and 48 h, followed by comparing their growth states in the R–B mixed juice by measuring the pH values and viable cell counts. The strain which could ferment the R–B mixed juice in a shorter time to reach a lower pH value and a higher viable cell count was selected as the final starter culture. The pH values were directly determined with a pH meter, and the viable cell count was recorded as colony-forming units (CFU) per mL.

### 2.3. Screening of Fermentation Conditions for L. plantarum TUST-232

The fermentation conditions of *L. plantarum* TUST-232 were screened in terms of different inoculation volumes (0.03%, 0.06%, 0.10%, 0.20%, 0.30%, and 0.40% with separate trials at 37 °C and fermented for 14 h), fermentation temperatures (30 °C, 34 °C, 37 °C, and 40 °C with separate trials with 0.30% inoculation volume and fermented for 14 h), and fermentation time periods (10 h, 12 h, 14 h, 16 h, and 18 h with consecutive trials under different time points with 0.30% inoculation volume and fermented at 37 °C). The fermentation ability of *L. plantarum* TUST-232 was measured in terms of pH value and viable cell count, and the final inoculation volume, fermentation temperature, and fermentation time of the R–B mixed juice were determined by the lower pH value and the higher viable cell count [14,15]. After screening, the growth curve of *L. plantarum* TUST-232 was determined under the screening conditions [16] and measured in terms of pH value, total titratable acidity (TTA) value, and viable cell count. The TTA value was determined by titrating 1 mL of R–B mixed juice with 0.1 M NaOH at 22 °C.

### 2.4. Changes in Sugar Content

The contents of sucrose, glucose, and fructose in the R–B mixed juice before and after fermentation were analyzed as described by Baiquan et al. [17] and Pereira et al. [18] with slight modifications. The R–B mixed juice before and after fermentation was fivefold diluted with ultrapure water and centrifuged at 10,200× *g* for 5 min. The supernatants were separated and filtered through a syringe filter with 0.22 μm pore size and 13 mm diameter membrane Millipore^®^ (Millipore, Billerica, MA, USA). The separation for identification of sugars were carried out by a high-performance liquid chromatograph (HPLC) using an RID-10A detector (Shimadzu, Japan) and an Agilent^®^ Hi-Plex Ca (Duo) (300 mm × 6.5 mm) chromatographic column. The column temperature was maintained at 65 °C, and the injection volume was 10 μL. Ultrapure water was used as the mobile phase at a flow rate of 0.5 mL/min. The contents of sugars were quantified by an external standard method. Standard solutions of sucrose, glucose, and fructose (SolarBio, Beijing, China) were diluted to different concentrations (0.5, 1, 2.5, 5, and 10 mg/mL), and standard curves were obtained according to the concentration and the corresponding peak area. The peaks of sugars were determined by reference to the retention times of standard solutions, and the contents of sucrose, glucose, and fructose in the R–B mixed juice before and after fermentation were calculated by interpolation of the peak areas.

### 2.5. Changes in Organic Acid Content

The contents of oxalic acid, citric acid, malic acid, succinic acid, lactic acid, and adipic acid before and after fermentation were analyzed as described by Zheng et al. [19] with slight modifications. The R–B mixed juice before and after fermentation was 50-fold diluted with ultrapure water and centrifuged at 10,200× *g* for 5 min. The supernatants were separated and filtered through a syringe filter with a 0.22 μm pore size and 13 mm diameter membrane Millipore^®^ (Millipore, Billerica, MA, USA). The separation for identification of organic acids was carried out by an HPLC with an LC-20A detector (Shimadzu, Japan) and Agilent^®^ Hi-Plex H chromatographic column (300 mm × 7.7 mm), and the detector was operated at 210 nm [20]. The injection volume of the treated sample was 20 μL, and the column temperature was maintained at 65 °C. Dilute sulfuric acid solution (0.05 mol/L) was used as the mobile phase at a flow rate of 0.5 mL/min. The contents of organic acids were quantified by an external standard method. The standard solutions of oxalic acid, citric acid, malic acid, succinic acid, lactic acid, and adipic acid (SolarBio, Beijing, China) were diluted to different concentrations (12.5, 25, 50, 75, and 100 μg/mL), and the standard curves were obtained according to the concentration and the corresponding peak area. The peaks of organic acids were determined by reference to the retention times of standard solutions, and the contents of oxalic acid, citric acid, malic acid, succinic acid, lactic acid, and adipic acid in the R–B mixed juice before and after fermentation were calculated by interpolation of the peak areas.

### 2.6. GC–MS Analysis of Volatile Compounds

Headspace solid-phase microextraction (HS-SPME) was used to prepare the volatile compounds before and after fermentation [12]. The volatile compounds were analyzed by a QP2010 Ultra gas chromatograph–mass spectrometer (Shimadzu, Japan) with a BR-SWAX (30 m × 0.32 mm × 0.50 μm) quartz capillary column. Briefly, the R–B mixed juice before and after fermentation was poured into sample bottles and heated at 55 °C for 30 min, followed by treating the SPME fiber assembly (PDMS/DVB 65 μm) at 250 °C for 30 min in the inlet, and then the adsorption of the gas by the fiber for 30 min. Subsequently, the SPME fiber was analyzed at 250 °C for 15 min, and the volatile compounds were further analyzed for 30 min after removing the fiber. In the GC conditions, the sample was split in split mode with a 10:1 split ratio, and the carrier gas was high-purity helium at 1 mL/min, while the inlet temperature was 240 °C. The initial temperature of the column was maintained at 50 °C for 4 min and then increased to 150 °C at 4 °C/min, with each temperature point maintained for 3 min. Finally, the temperature was raised to 250 °C at 8 °C/min, with each temperature point held for 5 min. In the MS conditions, the ionization mode was EI, the electric energy was 70 eV, the heating current of the filament was 0.25 mA, the voltage of electronic multiplier was 1000 V, the temperature of the ion source was 220 °C, the transmission line temperature was 220 °C, and the scanning range was 40–500 (*m/z*).

The results of determination obtained were cross-referenced with the NIST 05 library, and the relative content of each volatile compound was measured by the peak area normalization method. The volatile components with the highest peak area in the top 70 and a high matching degree were selected.

### 2.7. Electronic Nose Analysis

The changes in the aroma profile of the R–B mixed juice before and after fermentation were detected by a portable electronic nose (PEN 3.5 Portable Electronic nose, Airsense Analytics GmbH, Schwerin, Germany) and analyzed as described by Wang et al. [21] with slight modifications. The electronic nose, an electronic system that imitates the human olfactory system and uses a gas sensor array to identify odor, inhales the aroma profile of the sample to be tested into the instrument and analyzes the composition of the gas components through 10 metal–oxide semiconductor (MOS) sensors (R1, W1C, aromatic; R2, W5S, broad-range; R3, W3C, aromatic; R4, W6S, hydrogen; R5, W5C, arom-aliph; R6, W1S, broad-methane; R7, W1W, sulfur-organic; R8, W2S, broad-alcohol; R9, W2W, sulf-chlor; R10, W3S, methane-aliph) [22]. Before the test, 10 mL of R–B mixed juice before and after fermentation was transferred separately to a 50 mL beaker and sealed with plastic wrap, followed by equilibration for 30 min at room temperature (20–25 °C). During the test, the cleaning time and detection time of the instrument were set at 120 s and 100 s, respectively. Lastly, the results were analyzed using WinmMuster version 1.6.2.18 software (Airsense Analytics GmbH, Schwerin, Germany).

### 2.8. Antioxidant Activity and the Contents of Total Phenolic, Total Flavonoid, and Dietary Fibers

The total antioxidant capacity was determined using the ferric reducing ability of plasma (FRAP) assay in accordance with the instructions of the T-AOC Kit (SolarBio, Beijing, China). Before the test, the R–B mixed juice before and after fermentation was centrifuged at 2500× *g* for 10 min to collect the supernatant for FRAP assay. The absorbance was determined at 593 nm, and the results were expressed as μmol Fe^2+^ equivalents/100 mL [23].

The iron ion-reducing ability was determined by the Prussian blue method using the Texting Kit (Congyi Biology, Shanghai, China) [24]. The reduction ability of iron ions was expressed by the absorbance value at 700 nm, whereby a larger absorbance value denoted a stronger reduction ability of the R–B mixed juice to iron ions. The supernatant was ninefold diluted with ultrapure water for Prussian blue analysis, and the antioxidant Trolox (0.04 mM) was used as a positive control.

The hydroxyl radical-scavenging activity was determined using the Micro Hydroxyl Free-Radical Scavenging Capacity Assay Kit (SolarBio, Beijing, China). The inhibition degree of the 536 nm absorbance decline rate (%) of the R–B mixed juice was used to reflect its ability to scavenge hydroxyl radicals [25]. Ascorbic acid (0.075 mg/mL) was used as a positive control.

The superoxide anion-scavenging ability was determined using the Inhibition and Produce Superoxide Anion Assay Kit (Jiancheng Bioengineering Institute, Nanjing, China). The scavenging ability was expressed by the absorbance value at 550 nm, whereby a lower absorbance value denoted a stronger scavenging ability of the R–B mixed juice to superoxide anion. The superoxide anion scavenging ability of the R–B mixed juice was calculated with ascorbic acid as the standard (U/100 mL).

To determine total phenolic content, the R–B mixed juice before and after fermentation was ninefold diluted with ultrapure water, followed by placing 2 mL of the diluent and 1 mL of Folin–phenol solution in a volumetric flask. After incubation for 6 min, the mixture was supplemented with 1 mL of 20% sodium carbonate solution and incubated at 30 °C for 1 h. The absorbance was determined at 760 nm using a UV/visible spectrophotometer, with distilled water as the blank control. Finally, the total phenolic content was calculated on the basis of a gallic acid calibration curve (*R^2^* = 0.9996) [26,27,28].

To determine total flavonoid content, the R–B mixed juice before and after fermentation (1 mL) was supplemented with 0.5 mL of sodium nitrite solution (5 g/100 mL) in a volumetric flask, followed by incubation for 6 min and the addition of 0.5 mL of aluminum nitrate solution (10 g/100 mL). After incubation for 6 min, the mixture was supplemented with 4 mL of sodium hydroxide solution (4 g/100 mL) and then thoroughly mixed for 1 min. The absorbance was determined at 510 nm using a UV/visible spectrophotometer, with distilled water as the blank control. The total flavonoid content was calculated on the basis of a rutin calibration curve (*R^2^* = 0.9989) [29].

The contents of total, insoluble, and soluble dietary fibers in the R–B mixed juice before and after fermentation were determined by the AOAC methods [30].

### 2.9. Color Measurement

The changes in color of the R–B mixed juice before and after fermentation were detected by a chromatometer (CM-5; MINOLTA, Japan) [31,32]. According to the definition, the color of the sample is defined by three-coordinate space values: the L* (lightness) value, a* (red/green component) value, and b* (yellow/blue component) value. The changes in the sample color before and after fermentation were analyzed by the following equation:

ΔE^*^_a__b_ = [(L* − L_0_*)^2^ + (a* − a_0_*)^2^ + (b* − b_0_*)^2^]^1⁄2^, (1)

where ΔE*_ab_ indicates the change in color, L_0_* and L* indicate the lightness value before and after fermentation, respectively, a_0_* and a* indicate the red/green component value before and after fermentation, respectively, and b_0_* and b* indicate the yellow/blue component value before and after fermentation, respectively.

### 2.10. Statistical Analyses

All the experiments were repeated three times. The values of pH, TTA, and viable cell counts were treated by one-way analysis of variance (ANOVA). The values of sugar content, organic acid content, volatile compound content, total phenolic content, total flavonoid content, antioxidant activity, and dietary fiber content were treated by a two-tailed paired Student’s *t*-test. The significant difference for each comparison was determined at *p* < 0.05. Data were expressed as the mean ± standard deviation (SD).

## 3. Results

### 3.1. Selection of Starter Culture and Fermentation Conditions

#### 3.1.1. Selection of Starter Culture

A comparison of the fermentation ability to the R–B mixed juice was made for different LAB strains, including mixed *L. delbrueckii* subsp. *bulgaricus* and *S. thermophilus* (TUST-BS for short), *L. plantarum* TUST-232, *L. plantarum* TUST-354, and *L. plantarum* TUST-232. Due to their symbiotic relationship in the exchange of metabolites, *L. delbrueckii* subsp. *bulgaricus* and *S. thermophilus* were mixed before fermentation [33]. A comparison of the pH values and the viable cell counts in the R–B mixed juice revealed the *L. plantarum* TUST-232 strain as the most suitable strain for fermentation (Table 1), with the viable cell count reaching 8.91 log CFU/mL after fermentation for 24 h, which was significantly higher (*p* < 0.05) than that of the other strains, in contrast to the lowest value of 3.10 log CFU/mL in the R–B mixed juice fermented by *L. plantarum* TUST-392 under the same conditions. The pH value decreased to 3.43 in the R–B mixed juice fermented by *L. plantarum* TUST-232, which was significantly lower than that fermented by the other strains. After 48 h of fermentation, the viable cell count reached 7.83 log CFU/mL in the TUST-BS fermented R–B mixed juice, and the pH values of all trial groups decreased to less than 3.80. However, *L. plantarum* TUST-232 was still higher (*p* < 0.05) than the other strains in the viable cell count in the R–B mixed juices, and, compared with *L. plantarum* TUST-232, the other strains had a more extended lag phase and required a longer time to enter the log phase. Therefore, *L. plantarum* TUST-232 was determined as the final starter culture for further experiments.

#### 3.1.2. Selection of Fermentation Conditions

**Figure 1:**
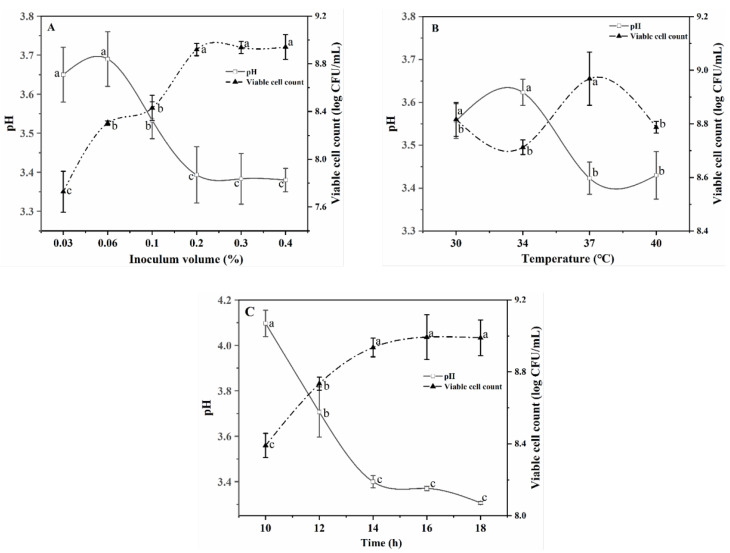
Changes in pH values and viable cell counts under different inoculation volumes (**A**) (0.03%, 0.06%, 0.10%, 0.20%, 0.30%, and 0.40% with separate trials at 37 °C and fermented for 14 h), fermentation temperatures (**B**) (30 °C, 34 °C, 37 °C, and 40 °C with separate trials with 0.30% inoculation volume and fermented for 14 h), and fermentation time periods (**C**) (10 h, 12 h, 14 h, 16 h, and 18 h with consecutive trials under different time points with 0.30% inoculation volume and fermented at 37 °C) with *L. plantarum* TUST-232. ^a,b,c^ Statistical analysis ANOVA at 95% confidence level, with different letters indicating a significant difference at *p* < 0.05.

Under different inoculation volumes of *L. plantarum* TUST-232, the R–B mixed juices varied in their pH values and viable cell counts (Figure 1A). At an inoculation volume less than 0.2%, the organic acids produced by *L. plantarum* TUST-232 were positively correlated with the inoculation volume, in contrast to no significant difference among indicators related to fermentation performance at an inoculation volume above 0.2%. Under the three inoculation volumes (0.2%, 0.3%, and 0.4%), *L. plantarum* TUST-232 in the R–B mixed juices all entered the stationary phase within 14 h. A higher inoculation volume can avoid the contamination of the R–B mixed juice caused by pathogenic microorganisms in the environment in the early stage of fermentation, while it can also produce more volatile compounds during the fermentation [34]. Therefore, the inoculation volume was selected as 0.3% for further experiments. The pH values and viable cell counts under different fermentation temperatures were compared to determine the fermentation temperature. When fermented at 37 °C, the R–B mixed juice showed the lowest pH value and highest viable cell count of 3.42 and 8.91 log CFU/mL, respectively (Figure 1B), in contrast to a higher pH value and lower viable cell count at a temperature lower or higher than 37 °C. Therefore, 37 °C was determined as the final fermentation temperature for further experiments.

In Figure 1C, the pH values of the fermented R–B mixed juice were shown to decrease gradually with the prolongation of fermentation time. After 14 h, the pH values began to decline slowly, and the growth of *L. plantarum* TUST-232 began to slow down, entering the stationary phase. Therefore, 14 h was defined as the final fermentation time.

#### 3.1.3. Growth Curve of *L. plantarum* TUST-232

**Figure 2:**
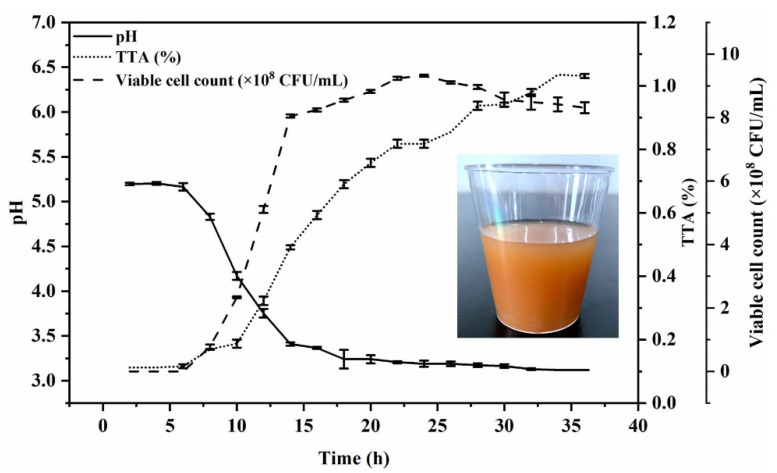
Changes in viable cell counts, pH values, and TTA values during fermentation of the R–B mixed juice with *L. plantarum* TUST-232, and the picture of the beverage produced under the selected fermentation conditions. The growth curve was determined under the selected conditions of 0.3% inoculation volume and fermentation at 37 °C for 36 h.

To determine the growth trend of the strain with a longer fermentation time, Figure 2 shows the growth curve and the change trend of pH values and TTA values from 0–36 h under the selected conditions of 0.3% inoculation volume and fermentation at 37 °C. *L. plantarum* TUST-232 entered the logarithmic growth phase at 6 h, and viable cell counts increased exponentially between 6 and 14 h, followed by a plateau and then a slow down after 24 h.

Meanwhile, the pH values in the R–B mixed juice decreased from 5.2 to 3.1 from 0 to 36 h, with an obvious drop from 6 to 14 h, followed by a slow decrease.

Furthermore, due to the continuous growth of *L. plantarum* TUST-232, the TTA value showed a rapid increase from 6 h onward, reaching 0.49% at 14 h of fermentation. At the time beyond 14 h, the TTA value maintained a steady increase. Accordingly, we developed the fermented beverage of red jujube fruits and bamboo shoots under the selected conditions (Figure 2).

### 3.2. Changes in Sugar Content

**Figure 3:**
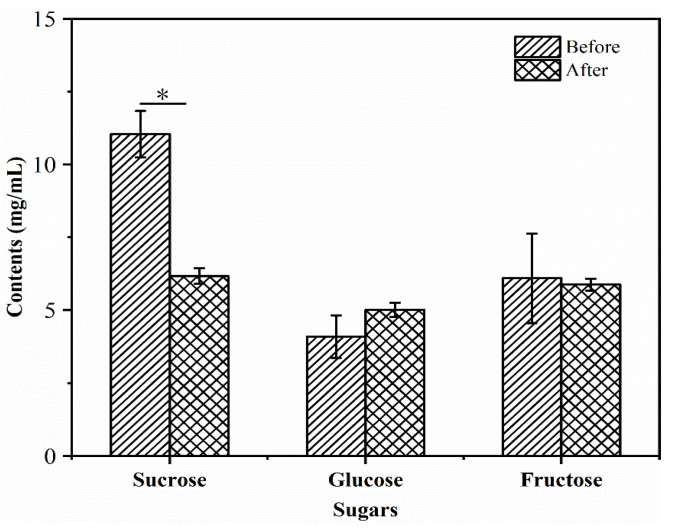
Changes in the content of sugars before and after fermentation of the R–B mixed juice with *L. plantarum* TUST-232. The contents of sugars, including sucrose, glucose, and fructose, were measured. The error bars indicate means ± SD (*n* = 3); the differences between groups were analyzed by a two-tailed paired Student’s *t*-test; * *p* < 0.05.

During fermentation, the contents of sugars in the R–B mixed juice could meet the growth requirement of *L. plantarum* TUST-232. The changes in glucose, fructose, and sucrose content in the R–B mixed juice are shown in Figure 3, and the *R^2^* values of the calibration curves were 0.9987, 0.9995, and 0.9996, respectively. Among the three sugars, sucrose was most abundant in the R–B mixed juice before and after fermentation, with the highest consumption rate of 44.10% during fermentation (*p* < 0.05). After fermentation, the contents of fructose and glucose exhibited no significant change in the R–B mixed juice.

### 3.3. Changes in Organic Acid Content

**Figure 4:**
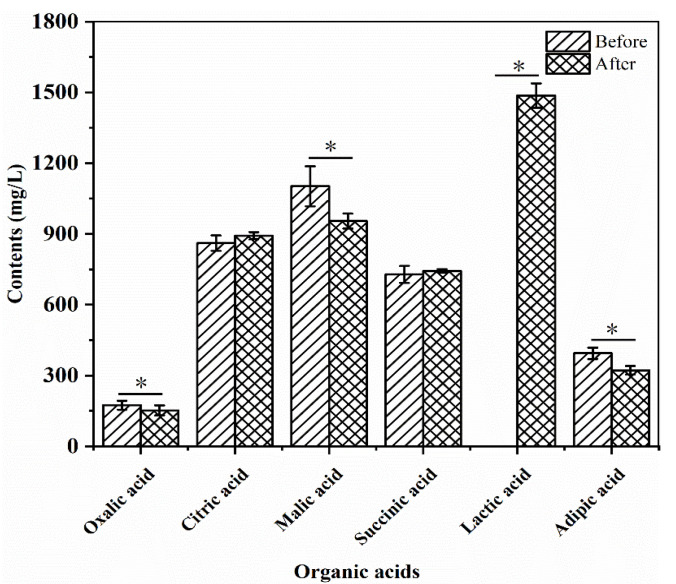
Changes in the content of organic acids before and after fermentation of the R–B mixed juice fermented with *L. plantarum* TUST-232. Organic acids, including oxalic acid, citric acid, malic acid, succinic acid, lactic acid, and adipic acid, were measured. The error bars indicate means ± SD (*n* = 3); the differences between groups were analyzed by a two-tailed paired Student’s *t*-test. * indicates significant differences at *p* < 0.05.

The changes in the contents of oxalic acid, citric acid, malic acid, succinic acid, lactic acid, and adipic acid in the R–B mixed juice before and after fermentation with *L. plantarum* TUST-232 are shown in Figure 4, and the *R^2^* values of the calibration curves were 0.9997, 0.9972, 0.9999, 0.9999, 0.9999, and 0.9997, respectively. Before fermentation, the content of malic acid was significantly higher (*p* < 0.05) than that of other organic acids in the R–B mixed juice. Despite a decrease of 13.41% after fermentation, the malic acid content was still second only to lactic acid. Meanwhile, the contents of oxalic acid and adipic acid showed decreases of 12.61% and 18.25% after fermentation (*p* < 0.05), respectively, despite their lowest content in the R–B mixed juice. Furthermore, the contents of citric acid and succinic acid had no significant change after fermentation, and lactic acid was not detectable before fermentation, but its content reached 1.49 g/L after fermentation, which was the highest in all the measured organic acids.

### 3.4. Changes in Volatile Compounds

Table 2 shows the changes of volatile compounds in the R–B mixed juice before and after fermentation with *L. plantarum* TUST-232. Totals of 53 and 61 volatile compounds in the R–B mixed juice were detected before and after fermentation, respectively, including 16 aldehydes, two olefins, 10 ketones, eight alcohols, three esters, 11 acids, and three other volatile compounds before fermentation, as well as 16 aldehydes, four olefins, eight ketones, 13 alcohols, six esters, 11 acids, and three other volatile compounds after fermentation. After fermentation, the R–B mixed juice showed a significant increase (*p* < 0.05) in the relative contents of some ketones, alcohols, and acids, such as 3-hydroxy-4-hexanone, 1-octen-3-one, heptanoic acid, nonyl alcohol, and myristic acid, in contrast to a significant decrease (*p* < 0.05) in the relative contents of some aldehydes, such as (2*Z*)-2-heptenal and heptanal. Furthermore, 16 flavor substances disappeared, and 24 flavor substances were newly produced.

### 3.5. Changes in Aroma Profile

**Figure 5:**
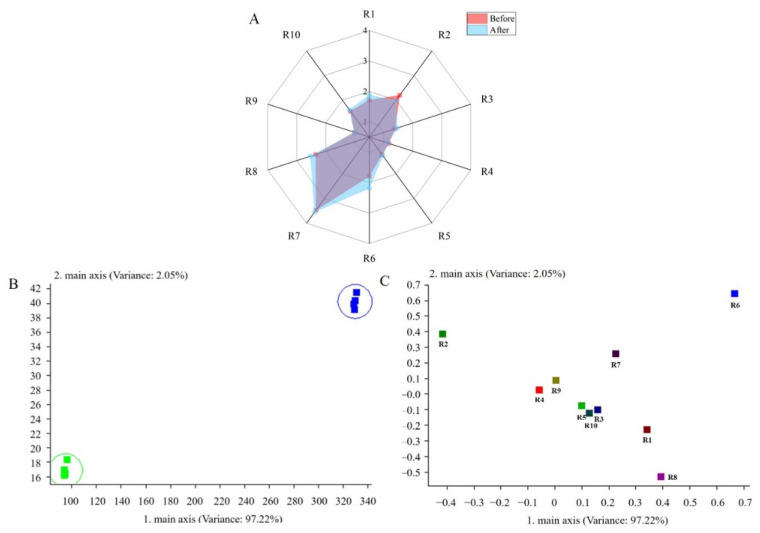
Radar plot (**A**), PCA score plot (**B**), and loading plot (**C**) of the aromatic compounds in the R–B mixed juice before and after fermentation detected by electronic nose.

The changes in the aroma profile of the R–B mixed juice before and after fermentation with *L. plantarum* TUST-232 are shown in Figure 5. The response values of the 10 sensors are reflected on a radar plot to show the changes in the aroma profile of the R–B mixed juice before and after fermentation (Figure 5A). The sensor response values of R1, R6, and R8 increased after fermentation, indicating that more aromatic compounds and alcohols were formed in the R–B mixed juice fermented by *L. plantarum* TUST-232. The decrease in R2 sensor response value indicated that nitrogen oxide was reduced after fermentation. Principal component analysis (PCA) revealed that the first two principal components (PC1, 97.22%; PC2, 2.05%) accounted for 99.27% of the total variance (Figure 5B,C). In Figure 5B, the aroma profile of the R–B mixed juice before and after fermentation was seen to be clearly distributed along PC1 and PC2, indicating an obvious difference in the aroma profile before and after fermentation. Figure 5C shows the PCA results regarding the contribution of the sensors to aroma profile identification in the R–B mixed juice before and after fermentation. The R–B mixed juice had a higher methane content before fermentation, making the S6 sensor more capable of identifying the odor before fermentation.

### 3.6. Changes of Antioxidant Activity and the Contents of Total Phenolic, Total Flavonoid, and Dietary Fibers

Compared with the unfermented R–B mixed juice, the fermented R–B mixed juice showed a significant improvement in total antioxidant capacity, iron ion-reducing ability, hydroxyl radical-scavenging activity, and superoxide anion-scavenging ability (Table 3). Specifically, after fermentation, the total antioxidant capacity increased by 12.30% and reached 94.743 μmol Fe^2+^ equivalents/100 mL; the absorbance of iron ion-reducing ability reached 0.315 versus the absorbance value of 0.297 for Trolox (0.04 mM); the hydroxyl radical-scavenging activity increased from 4.045% to 9.833% versus the scavenging activity of 16.667% for 0.075 mg/mL ascorbic acid; the superoxide anion-scavenging ability showed an increase of 59.80% and reached 7.629 U/100 mL.

Additionally, the R–B mixed juice fermented with *L. plantarum* TUST-232 showed a significant increase of 11.09% in total phenolic content and reached 32.918 mg/L after fermentation (Table 3), in contrast to no significant change in total flavonoid content.

Meanwhile, despite no significant changes in the contents of dietary fibers (Table 3), the R–B mixed juice fermented with *L. plantarum* TUST-232 still showed a higher content of soluble dietary fiber than beverages such as beers. The contents of total, insoluble and soluble dietary fiber reached 0.570 g/100 g, 0.492 g/100 g, and 0.078 g/100 g, respectively.

### 3.7. Changes in Color

The changes in the color of the R–B mixed juice before and after fermentation were analyzed by L* value, a* value, and b* value, and the results are shown in Table 4. After fermentation, the R–B mixed juice showed a decrease in brightness, as well as in red and yellow tones, but an increase in green and blue tones. During fermentation, nutrients were transformed to other related substances by *L. plantarum*, leading to changes in the color of the R–B mixed juice. The value of color ΔE*_ab_ was less than 1, indicating no significant change in the color between the fermented and unfermented R–B mixed juices, which was almost impossible to distinguish by the naked eye.

## 4. Discussion

In China, the yields of red jujube fruits and bamboo shoots are very high. Red jujube fruits are usually dried to extend their shelf life, but the extremely low moisture content greatly affects the taste of the dried red jujube fruits. Meanwhile, bamboo shoots are usually canned due to their short shelf life. These limitations greatly affect their off-season consumption and full utilization of nutritional values. In recent years, plant-based beverages, as an alternative to acid dairy products, have become more popular, and they provide an option for making full use of the nutrients of red jujube fruits and bamboo shoots in all seasons, suggesting the great potential of these two materials with a short shelf life for beverage development.

Red jujube is an excellent raw material for improving the flavor and antioxidant activity of the beverage, and bamboo shoot is rich in dietary fibers, thus being beneficial to the human intestines. Feng et al. [35] studied the effects of red jujube pulps on the quality characteristics and antioxidant activity of goat milk yogurt, and they found that adding red jujube pulps could significantly reduce the flavor, improve the sensory acceptance, and increase the antioxidant activity of yogurts. Nongdam and Tikendra [36] introduced the rich nutrients of bamboo shoot and considered it as a portion of health food. Eating foods high in dietary fibers can reduce harmful cholesterol in the blood, maintain digestive health, and improve constipation. The beverage prepared by combining the nutritional values of the two raw materials and the fermentation of *L. plantarum* will have potential market value.

Fermentation may be an effective method for inhibiting the growth of potentially pathogenic bacteria in foods, which can improve the safety of products. Canaviri, Jannat, and Håkansson [37] developed a quinoa fermented beverage using *L. plantarum* DSM 9843 and quinoa, with a significant decrease in the pH value of the beverage and an obvious increase in the content of lactic acid after 48 h of fermentation. In the present study, it was shown that *L. plantarum* TUST-232 can not only use the nutrients in the beverage for rapid growth, but also produce a large amount of lactic acid. Meanwhile, the final pH value of the beverage was also lower than 4, which is one of the crucial characteristics for the safety of a beverage [38]. When the pH value is lower than 4, the growth of pathogenic bacteria can be inhibited in the beverage, thereby extending shelf life and improving safety [39]. Although the lower pH value is beneficial to improve the shelf life of the beverage, it is necessary to adjust the sour taste in the beverage by adding sugars such as xylitol in the future, so that it can be accepted by more consumers.

Due to hydrolysis of glycan and oligosaccharide by enzymes secreted by *L. plantarum*, only sucrose, glucose, and fructose were selected for determination in the R–B mixed juice before and after fermentation in this study [40]. The viable cell count of the beverage fermented by *L. plantarum* TUST-232 could reach 8.91 log CFU/mL at 14 h, related to the high contents of natural sugars and in the R–B mixed juice. After fermentation, the apparent decrease in sucrose content can be attributed to the fact that sucrose was consumed by sucrose phosphorylase and acid hydrolysis produced by *L. plantarum* TUST-232, consistent with the observation of Lu, Putra, and Liu [41] in the fermentation of durian with LAB strains. LAB strains vary in their ability to consume sucrose, leading to changes in the content of sucrose before and after fermentation, thus affecting the fermentation of the strain and the viable cell count in the beverage. Glucose and fructose can be utilized as carbon sources for the growth of LAB, especially glucose [42]. After fermentation, the contents of glucose and fructose had no significant changes. Combined with the significant decrease in sucrose content, this phenomenon may be due to the decomposition rate of sucrose being equivalent to the consumption rate of glucose and fructose during the fermentation [40]. In addition, the contents of glucose and fructose had no significant changes, which may be related to the lower pH value and the significant decrease in the malic acid content of the R–B mixed juice after fermentation. It has been reported that, under low-pH fermentation conditions, *L. plantarum* would use a nonconventional carbon source such as malic acid and convert it into lactic acid, resulting in a significant decrease in sucrose content and no significant changes in glucose and fructose contents [43,44].

The starter plays an important role in the flavor of a fermented beverage, and *L. plantarum* TUST-232 was shown as a suitable starter for this fermented beverage, playing a key role in the metabolism of organic acids. Oxalic acid, citric acid, malic acid, succinic acid, lactic acid, and adipic acid are the primary organic acids in a beverage. During fermentation, *L. plantarum* TUST-232 can reduce the acidity of the beverage by converting malic acid into lactic acid, and the final content of malic acid was reduced by 13.41%. Jin et al. [45] used six different probiotics to ferment mango pulps, and LAB was shown to completely consume the malic acid in the mango pulps and convert it to lactic acid after fermentation. After fermentation by *L. plantarum* TUST-232, malic acid was converted into lactic acid, leading to a reduction in the acidity of beverage. At the same time, the flavor of the fermented beverage from the R–B mixture was also improved, related to the conversion of malic acid to lactic acid, resulting in a decrease of 13.41% in the final malic acid content. Zhang et al. [46] studied a mulberry beverage cocultured with *Saccharomyces cerevisiae* and *L. plantarum*. They found that *S. cerevisiae* and *L. plantarum* converted malic acid to lactic acid, which could not only reduce the acidity of beverage, but also improve the flavor through the generation of metabolites, thus enhancing the microbial stability and sensory quality of the final product. Meanwhile, some oxalic acid and adipic acid in the beverage could be metabolized by *L. plantarum* as carbon and energy sources. During 48 h fermentation, the content of citric acid showed a continuous increase from 0–24 h, followed by a sharp drop. *L. plantarum* TUST-232 can produce succinic acid instead of lactic acid, and the final content of succinic acid was increased by 2.08%.

Flavor is one of the essential properties of a fermented beverage, and the peak area normalization method was used calculate the relative content of the volatile compounds. The pre-fermented beverage was relatively simple in flavor, mainly involving the smell of red jujube fruits and bamboo shoots. The fermentation of *L. plantarum* TUST-232 released and produced some volatile compounds, such as acids, aldehydes, ketones, and other substances, thus improving the quality and flavor of the beverage. After fermentation by *L. plantarum* TUST-232, the R–B mixed juice showed great changes in the composition and relative content of some main volatile compounds, such as hexanal, (2*Z*)-2-heptenal, 2-octenal, benzaldehyde, 1-octen-3-one, hexanoic acid, and decanoic acid. These compounds have been identified as important volatile compounds in the foods fermented by *Lactobacilli* [47]. Among the main volatile compounds, due to the fermentation of *L. plantarum* TUST-232, the relative contents of acids and aldehydes were 31.61% and 42.27%, respectively, and they played a key role in the characteristic flavor of the R–B mixed juice.

Previous studies showed that the relationship between the volatile compounds and fermentation substrates was complex [48]. The fermentation of *L. plantarum* TUST-232 increased the content of some organic acids, which not only improved the acidity of the beverage, but also changed the relative contents of some volatile compounds. Malolactic fermentation (MLF) is a process via which *L. plantarum* converts malic acid into lactic acid and carbon dioxide through the secretion of the malolactic enzyme (MLE). After 14 h of fermentation, the content of malic acid in the mixed juice decreased significantly and the content of lactic acid increased significantly, indicating that the MLE secreted by *L. plantarum* TUST-232 was highly active in performing MLF during the fermentation [49]. At the same time, MLF made it possible for *L. plantarum* to grow well without adding nutrients before fermentation. In addition, the MLF can improve the flavor and taste of beverages, produce many volatile secondary compounds, and improve the stability [50]. Lactic acid participated in ester metabolism during the fermentation, which led the formation of some esters. After fermentation, the relative content of olefins decreased by 17.13%, which was related to the participation of malic acid in the metabolic pathway of olefins [48]. The relative content of acetic acid increased significantly after fermentation, indicating that *L. plantarum* TUST-232 activated the acetate kinase route of the phosphogluconate pathway [51]. *L. plantarum* could consume lactic acid and release the same amount of acetic acid under aerobic conditions [47].

After fermentation, the R–B mixed juice showed great changes in the relative contents of several main aldehydes, such as the disappearance of furfural and heptanal, which could help to reduce the “caramel-like” flavor and the strong fruity odor of the beverage, respectively [52]. Meanwhile, a significant increase was observed in the relative contents of 2-octenal and 2-undecenal; the former has the aromas of fat, meat, cucumber and chicken, while the latter has a strong aroma of unsaturated aldehyde fragrance, both of which may improve the flavor of the beverage.

The relative contents of ketone, ester, and alcohol also varied in their effects on the flavor of the beverage. After fermentation, some ketones could synergistically enhance the fruity aroma of the fermented beverage [45]. The beverage contained some fatty acids, such as methyl ketones (2-nonanone, 2-octanone), which could have been formed by β-oxidation of fatty acids and decarboxylation of the produced β-keto acids [47]. Before fermentation, 6,10-dimethyl-5,9-undecadien-2-one was not detectable, but it was detected to reach a certain amount after fermentation, endowing the beverage with a sweet rose aroma. The formation of esters was mainly related to the consumption of sugars by *L. plantarum* [53]. Due to a lower odor threshold of esters than alcohols, the esters in the beverage after fermentation may produce a great impact on the aroma of the beverage, and the relatively low contents of esters such as methyl laurate, decyl propionate, and benzyl salicylate may have positive effects on the beverage flavor [46]. Meanwhile, the formation of esters was also related to the abundance of alcohols in the beverage. During fermentation, *L. plantarum* produced many alcohols as precursors for esters [54], and, after fermentation, farnesol, nonyl alcohol, and benzyldehyde further released the plant aroma of the beverage, contributing to the flavor of the beverage. Furthermore, heterocyclic compounds such as 5-hydroxymethyl-2-furaldehyde were detected before and after fermentation, probably due to the heating of sugars and the production of some new substances [55]. Hexanal with a unique herbal flavor is the most reducing volatile compound and can reduce the strong plant odor of the beverage [56].

The primary antioxidants in red jujube fruits and bamboo shoots are mainly phenolic substances and flavonoid compounds. After fermentation, the R–B mixed juice showed no significant change in total flavonoid content, but an increase in total phenolic content, probably due to a low metabolic capacity of *L. plantarum* TUST-232 for flavonoid compounds in the R–B mixed juice [57]. The removal of the shells from bamboo shoots before fermentation to reduce their effect on the taste of the beverage resulted in a lower total phenolic content in the R–B mixed beverage. Katina et al. [58] reported that fermentation of rye bran by LAB could increase the total phenolic content by 90%, in contrast to an increase of only 15–30% for the fermentation of peeled bran, indicating that the total phenolic content in bran and some food shells may account for a certain proportion. After fermentation, the total phenolic content showed an increase of 11.09% in the beverage, which was related to the conversion and depolymerization of related compounds by enzymes, suggesting that *L. plantarum* TUST-232 can produce related enzymes in the beverage during fermentation to increase the total phenolic content by decomposing hydroxyl or hydroxyl groups in the phenol ring and producing phenolic acids [9,27,59]. In addition, the fermentation time of *L. plantarum* TUST-232 also played an important role in total phenolic content, and the fermentation conditions provided sufficient time for *L. plantarum* TUST-232 to increase the total phenolic content in the R–B mixed juice. Sidari et al. [60] evaluated the effect of the multi-strain starter culture (two LAB strains and a yeast strain) on the antioxidant and rheological properties of sourdoughs and derived bread. They found that the total phenolic content in the sourdough inoculated with LAB and yeast increased with the extension of fermentation time, resulting in significantly higher total phenolic content than that of the control group. Since the Folin–C method can measure all the phenolic substances, including aromatic amino acids, the increase in free phenolic can also be attributed to the increased protein hydrolysis during fermentation [58].

After fermentation, all four antioxidant indices (Table 3) showed significant improvement, probably due to the high ability of *L. plantarum* TUST232 to reduce iron ions, scavenge hydroxyl radicals, and inhibit superoxide anions, as well as its strong capability to hydrolyze polyphenols in the R–B mixed juice. Di et al. [61] used eight LAB strains to ferment *Portulaca oleracea* L. juice separately, and they found that all LAB strains could significantly improve several antioxidant indices of the juice. The improvement in antioxidant indices caused by LAB may also be related to various metabolites with antioxidant activity, such as glutathione, butyrate, and folic acid [62]. Therefore, the enhanced antioxidant capacity may be closely related to the unique biological activity and metabolic characteristics of *L. plantarum* TUST-232.

Most of the dietary fibers in the R–B mixed juice were insoluble or slowly fermented fibers, resulting in no significant change in the content of dietary fibers after fermentation [63,64]. Contemporary research has shown that most noncommunicable diseases are caused by an unhealthy diet and lifestyle, and new dietary habits are likely to cause intestinal microbial disorders. Ancestral diets are rich in plant-based foods and dietary fibers and are beneficial to ecological diversity, making the enteric microorganism more abundant and complex, thereby facilitating body metabolism and mental health [65]. A daily intake of 25–29 g dietary fibers can significantly reduce the risk of a series of diseases [66], and the content of dietary fibers in the fermented beverage could reach 5.9 g per kilogram. Furthermore, the beverage contained 0.78 g of soluble dietary fiber per kilogram, which is high relative to some other beverages like beer (0.2 g/L) and white wine (0.19 g/L) [67]. Therefore, this fermented beverage is a good choice for people with a low intake of daily dietary fibers.

## 5. Conclusions

A fermented beverage was prepared using red jujube fruits and bamboo shoots high in yield but difficult to store. *L. plantarum* TUST-232 fermentation changed the physicochemical properties and nutrients of the beverage, leading to the production of a large amount of lactic acid and an increase in total phenolic content. Furthermore, the fermentation with this LAB also improved the antioxidant capacity of the R–B mixed juice.

## Figures and Tables

**Table 1:** Fermentation performance of different strains used in the R–B mixed juice.

Strain	24 h		48 h	
	pH	Viable Cell Count (Log CFU/mL)	pH	Viable Cell Count (Log CFU/mL)
TUST-BS	4.17 ± 0.08 ^b^	3.41 ± 0.11 ^c^	3.51 ± 0.05 ^b^	7.83 ± 0.11 ^c^
TUST-232	3.43 ± 0.19 ^a^	8.90 ± 0.10 ^a^	3.29 ± 0.06 ^a^	8.69 ± 0.19 ^a^
TUST-392	4.7 ± 0.16 ^c^	3.10 ± 0.29 ^c^	3.79 ± 0.12 ^c^	4.74 ± 0.08 ^d^
TUST-354	3.99 ± 0.12 ^b^	4.64 ± 0.14 ^b^	3.31 ± 0.11 ^a^	8.42 ± 0.08 ^b^

^a,b,c^ Statistical analysis ANOVA at 95% confidence level: the value of TUST-232 in each column was defined as “^a^”; the closest value significantly different from “^a^” was defined as “^b^”; the next value significantly different from “^a^”, “^b^” and “^c^” was defined as “^d^”; different letters in each column indicate significant differences at *p* < 0.05.

**Table 2:** Main volatile compounds in the R–B mixed juice before and after fermentation with *L. plantarum* TUST-232.

Number	Name	Retention Time (min)	Relative Content (%)		*p*-Value
			Before	After	
	Aldehyde				
A1	3-methylbutyraldehyde	2.756	0.41	-	
A2	valeraldehyde	3.822	0.51	-	
A3	hexanal	6.125	6.16	2.38	*
A4	heptanal	8.827	3.35	-	
A5	5-methylhexanal	8.417	-	0.19	
A6	hexen-2-al	9.989	2.25	0.16	*
A7	octanal	11.926	3.14	2.81	
A8	(2*Z*)-2-heptenal	13.105	11.23	9.23	*
A9	nonanal	14.971	3.20	3.06	
A10	2-octenal	16.089	9.97	14.80	*
A11	furfural	17.106	0.91	-	
A12	decanal	18.367	-	0.67	
A13	lauric aldehyde	18.467	-	0.34	
A14	benzaldehyde	18.658	3.95	1.26	*
A15	2-nonenal	19.656	0.98	1.25	
A16	2-decenal	22.399	3.26	3.15	
A17	2,4-nonadienal	23.775	0.12	0.27	
A18	2-undecenal	24.929	1.25	2.41	*
A19	2-*trans*, 4-*trans*-decadienal	24.916	-	0.27	
A20	5-hydroxymethyl-2-furaldehyde	38.962	0.56	0.22	
	Olefin				
B1	2-methyl-6-methyl-2-methylene	15.227	-	0.91	
B2	3-ethyl-2-methyl-1,3-hexadien	15.659	0.08	0.25	
B3	2,4-dimethyl-1,3-pentadiene	17.160	-	0.58	
B4	3,5,5-trimethyl-2-Hexene	17.558	0.10	-	
B5	2-methyl-1,5-hexadiene	20.783	-	0.11	
	Ketone				
C1	3-hydroxy-4-hexanone	9.416	0.29	1.75	*
C2	3-octanone	10.917	0.13	-	
C3	1-octen-3-one	12.379	5.08	7.07	*
C4	6-methyl-5-hepten-2-one	13.457	0.47	0.89	*
C5	2-nonanone	14.883	0.11	0.37	*
C6	2-octanone	20.917	-	0.35	
C7	6-methyl-2-heptanone	23.618	0.33	-	
C8	l-fenchone	26.238	0.56	-	
C9	nerylacetone	26.913	-	0.59	
C10	6,10-dimethyl-5,9-undecadien-2-one	27.172	0.27	-	
C11	4,5-dimethyl-2-cyclohexen-1-one	30.164	0.48	-	
C12	3,6-dimethyl-5-octen-2-one	31.557	-	0.05	
C13	6,10-dimethyl-2-undecanone	33.092	0.40	0.46	
	Alcohol				
D1	3-octyn-2-ol	7.558	0.12	-	
D2	3,5,5-trimethyl-1-hexanol	7.857	-	0.16	
D3	amyl alcohol	11.201	0.08	-	
D4	hexyl alcohol	14.007	0.57	0.67	
D5	3,5-octadien-2-ol	15.500	0.16	0.15	
D6	1-octen-3-ol	16.623	2.97	1.55	*
D7	heptyl alcohol	16.775	-	0.33	
D8	1-octanol	19.821	0.72	0.71	
D9	(*E*)-2-octen-1-ol	21.215	1.59	0.92	*
D10	menthol	21.755	-	0.38	
D11	nonyl alcohol	22.198	-	1.21	
D12	nerolidol	26.048	-	1.30	
D13	benzyldehyde	27.112	-	0.58	
D14	farnesol	28.528	-	0.17	
D15	(2*E*,6*E*)-2,6-dimethyl-2,6-octadiene-1,8-diol	29.730	-	0.09	
D16	pentaethylene glycol	43.650	1.48	-	
	Ester				
E1	methyl benzoate	21.458	0.72	0.35	*
E2	geranyl phenylacetate	21.907	1.51	-	
E3	methyl Laurate	26.125	0.52	0.39	
E4	(*Z*)-3-decen-1-yl acetate	26.308	-	0.27	
E5	10-undecen-1-yl acetate	30.056	-	0.46	
E6	decyl propionate	35.042	-	0.30	
E7	benzyl salicylate	42.976	-	0.24	
	Acid				
F1	acetic acid	16.974	0.67	0.91	*
F2	valeric acid	24.010	-	0.03	
F3	hexanoic acid	26.500	0.77	1.80	*
F4	heptanoic acid	28.836	0.86	1.47	*
F5	octanoic acid	31.057	1.26	2.83	*
F6	nonanoic acid	33.382	0.69	0.88	
F7	decanoic acid	35.357	5.83	7.06	*
F8	undecanoic acid	37.183	0.18	0.14	
F9	lauric acid	38.739	6.37	8.72	*
F10	myristic acid	41.607	2.99	5.37	*
F11	palmitic acid	44.527	2.98	2.40	
F12	oleic acid	45.251	1.94	-	
	Others				
G1	2-pentylfuran	9.937	0.10	0.13	
G2	azulene	24.312	0.39	0.07	*
G3	adenosine cyclophosphate	29.060	0.08	-	
G4	2-methoxy-4-vinylphenol	37.310	-	0.35	

Relative content (%) represents the share of a given substance in the entire pool of volatile compounds; ‘before’ indicates the relative percentage in the R–B mixed juice before fermentation; ‘after’ indicates the relative percentage in the R–B mixed juice after fermentation; the differences between groups were analyzed by a two-tailed paired Student’s *t*-test; * *p* < 0.05.

**Table 3:** Changes in antioxidant activity and the contents of total phenolic, total flavonoid, and dietary fibers in the R–B mixed juice before and after fermentation with *L. plantarum* TUST-232.

Parameter	Before	After
Total antioxidant capacity (μmol Fe^2+^ equivalents/100 mL)	84.369 ± 1.891	94.743 ± 1.698 *
Iron ion-reducing ability (absorbance)	0.261 ± 0.009	0.315 ± 0.156 *
Hydroxyl radical-scavenging activity (%)	4.045 ± 0.639	9.833 ± 1.165 *
Superoxide anion-scavenging ability (U/100 mL)	4.774 ± 0.177	7.629 ± 0.292 *
Total phenolic (mg/L)	29.633 ± 1.075	32.918 ± 1.193 *
Total flavonoid (mg/L)	85.016 ± 4.321	95.810 ± 9.295
Total dietary fiber (g/100 g)	0.536 ± 0.024	0.570 ± 0.020
Insoluble dietary fiber (g/100 g)	0.472 ± 0.019	0.492 ± 0.021
Soluble dietary fiber (g/100 g)	0.059 ± 0.043	0.078 ± 0.017

The error bars indicate means ± SD (*n* = 3); the differences between groups were analyzed by a two-tailed paired Student’s *t*-test; * *p* < 0.05.

**Table 4:** Changes in color before and after fermentation of the R–B mixed juice with *L. plantarum* TUST-232.

Sample	L*	a*	b*	ΔL*	Δa*	Δb*	ΔE*_ab_
Before	38.47 ± 0.40	14.42 ± 0.19	45.00 ± 0.20	-	-	-	-
After	38.50 ± 0.19	14.32 ± 0.09	44.81 ± 0.12	0.03 ± 0.59	−0.10 ± 0.28	−0.19 ± 0.32	0.89

ΔE*_ab_, the change in color; L* (lightness); +L*, brighter; −L*, darker; a* (red/green component); +a*, redder; −a*, greener; b* (yellow/blue component); +b*, yellower; −b*, bluer. The error bars indicate means ± SD (*n* = 3); the differences between groups were analyzed by a two-tailed paired Student’s *t*-test.
